# Awareness of HIV/AIDS and its routes of transmission as well as access to health knowledge among rural residents in Western China: a cross-sectional study

**DOI:** 10.1186/s12889-019-7992-6

**Published:** 2019-12-04

**Authors:** Tianqi Zhang, Yang Miao, Lingui Li, Ying Bian

**Affiliations:** 1 Institute of Chinese Medical Sciences & State Key Laboratory of Quality Research in Chinese Medicine, University of Macau, Avenida da Universidade, Taipa, Macau, China; 2 College of Management, Ningxia Medical University, 1160 Shengli Street, Yinchuan, Ningxia Province China

**Keywords:** HIV/AIDS, Awareness rate, Transmission routes, Western China, Rural residents

## Abstract

**Background:**

The purpose of this study was to evaluate the coverage of HIV health education among rural residents in western China by ascertaining their awareness of HIV/AIDS and its transmission routes, and to investigate how these residents receive health information.

**Methods:**

A survey was conducted through stratified clustered sampling at 99 county hospitals in 11 provinces in western China. Information was collected on awareness of HIV/AIDS and its transmission routes, as well as residents’ access to health knowledge. Chi-square analysis was used to analyse the differences in HIV/AIDS awareness (knowing of the existence of HIV/AIDS, hereinafter referred to as “HIV awareness rate”) between different subgroups categorized by demographic status, regional factors, and different methods of access to health knowledge. To further analyse the effects of access to health knowledge on HIV awareness, a logistic regression model was established. The relationship between access to health knowledge and transmission routes was also examined using chi-square analysis.

**Results:**

The HIV awareness rate of the total 9274 participants was 80.9%. There were statistically significant differences between subgroups classified by age (*χ*^2^ = 482.118, *p*<0.001), education (*χ*^2^ = 853.465, p<0.001), occupation (*χ*^2^ = 340.553, p<0.001), income (*χ*^2^ = 186.448, *p*<0.001), cumulative HIV cases according to province (*χ*^2^ = 59.513, *p*<0.001), per capita annual net income of rural households according to province (*χ*^2^ = 64.676, *p*<0.001), proportion of minority population according to province (*χ*^2^ = 94.898, *p*<0.001), direct access to health knowledge (medical staff: *χ*^2^ = 419.775, *p*<0.001; mass media: *χ*^2^ = 740.238, *p*<0.001; family members: *χ*^2^ = 12.189, *p*<0.001; socializing: *χ*^2^ = 48.780, *p*<0.001; health education activities: *χ*^2^ = 154.400, *p*<0.001), and indirect access to health knowledge (having a non-communicable disease with medical instructions *χ*^2^ = 78.709, *p*<0.001; physical examinations: *χ*^2^ = 135.679, *p*<0.001). The logistic regression model showed that education and mass media had the strongest impacts on HIV awareness among all methods of access. Participants had the least awareness of HIV’s mother-to-child transmission route.

**Conclusion:**

The HIV awareness rate indicated that previous HIV health education covered 80% of the rural population in western China. Mass media should take greater responsibility in HIV health education for the general population, and special attention should be paid to the elderly, the most impoverished population, minority community as well as the mother-to-child transmission route.

## Background

The prevalence of HIV is a worldwide threat to public health. Although there is no cure for HIV, health education is considered as the most effective preventive measure. Being the most populous country, China places great importance on health education regarding HIV [1], and public education is one of the foundations of the Chinese HIV prevention and control system [1, 2].

Since the first case was diagnosed in Beijing in 1985, the Chinese government added requirements for health education to all national planning documents. However, it was not until the outbreak of HIV/AIDS among intravenous drug users (IDU) in Yunnan, a southwestern province, around 1990, that HIV awareness started to grow. In the mid-1990s, the HIV epidemic among commercial plasma donors in the central provinces was exposed. At around the same time, the central government published the first outline for HIV/STD health education, requiring local governments to provide HIV health education for the general population. In 1998, the government established basic principles for HIV/STD health education, and published an official handbook of basic information on HIV/AIDS [3]. In this way, China built up a clear policy framework for HIV health education.

Before 1998, HIV health education in China was mainly motivated by a political responsibility to respond to World AIDS Day [1, 4]. There were also disagreements about how to facilitate condom use and sexual education [5]. After 1998, the transportation, education, public security, and postal service departments, the entertainment sector, and NGOs began to play active roles in this field [3]. The central government’s 2004 HIV education plan recognized that rural areas still lacked HIV knowledge, and called for attention to rural areas, especially minority communities [6]. In response, a manual written in five ethnic languages was published, and clergy members started engaging in HIV health education among minority communities [7, 8].

In China, one outcome of the longstanding separation between urban and rural areas is the inequality of health services [9]. The rapidly developing economic process of urbanization drives thousands of villagers into the cities. Each year, a massive migrant population mobilizes in a country-city-country cycle. Part of this “floating population” engages in commercial sex, either as commercial sex workers (CSW) or paying customers [10]. Thus, they become a bridge population carrying HIV from high-prevalence cities to rural areas [10, 11]. More than half of those with newly diagnosed HIV in 2015 were from underdeveloped rural areas [12], and knowledge of HIV was lower in these areas [11, 13]. For these people, poor knowledge is a key factor in exposure to HIV infection and transmission [11, 14]. According to knowledge-attitude-practice (KAP) theory, knowing the existence of a disease and acquiring accurate knowledge is the foundation of health behaviour. In this context, there is a need for China to provide more HIV health education for residents of underdeveloped rural areas after getting a grasp on the effects of existing HIV health education.

On the other hand, previous research on HIV knowledge might have led to an overestimation of the effects of HIV health education [13, 15]. This overestimation could be attributed to the phenomenon of previous researchers inviting participants to fill the official questionnaire on HIV knowledge without asking whether they knew what HIV was. Therefore, some participants may just become familiar with HIV while reading the questionnaire and, if they select the correct answers, they may be assessed as having knowledge of HIV. This might contribute to a higher rate of reported HIV awareness, especially for rural areas [15]. Therefore the lack of accurate data had to be compensated.

The aim of this study was to evaluate the coverage of HIV health education in rural China by ascertaining the awareness of HIV/AIDS and its transmission routes among local residents, and to further investigate how these residents receive health information. Geographically, China can be divided into the eastern, central, and western areas. Socioeconomic development of the western area has lagged far behind since the Chinese reform and opening in 1978 [16]. In 2000, China initiated the Western Development Program (WDP), which ultimately established “western China” as a political term for an area consisting of 12 provincial divisions. It is a vast area, having more than fifty minorities living together with the Han people. The natural environment, and cultural and socioeconomic conditions, are more complex in the western part of the country than in the eastern and central areas, and these factors make health education more complicated [17]. Administratively, western China could also be categorized into two sections under a historical urban-rural dual structure. The rural area of western China is the least developed area. We conducted this research in rural western China.

## Methods

### Study area and setting

The study was conducted in 11 provincial administrative divisions (hereinafter “provinces”) in western China, in December 2011, as shown in Table 1. Western China covers 72% of the country’s territory, for a total of 6.86 million square kilometres. Most of this area consists of highland which is more than 1000 m above sea level. This area has a border approximately 20 thousand kilometres long and is contiguous to 10 countries, where two of the world’s main drug resources are located - the Golden Triangle and the Golden Crescent. It includes 6 provinces, 5 minority autonomous regions, and 1 municipality. Previously, Yunnan, Guangxi, and Sichuan were ranked among the top five provinces with newly diagnosed HIV cases via heterosexual transmission and intravenous drug injection [11, 12], and an outbreak of HIV also occurred in Xinjiang around 2000 [18]. One third of the Chinese population lives in this area (approximately 360 million people), and nearly 60% live in rural areas [19], including more than 50 minorities [16] with their own cultures, customs, and norms. Some also have unique languages and religions [20–22]. Religious influence is greater in this area than in the eastern or central areas – Islam forms a northwest-southeast belt extending from Xinjiang to Ningxia, whereas Buddhism forms a southwest-northeast belt extending from Tibet to Inner Mongolia [23]. Economic development, as a factor of HIV prevalence in Asian countries, is disrupting traditional society [24]. For better economic status, a large rural population moves inside this area as well as between other parts of China [11, 14, 16]. Additionally, drug trafficking, which originates in the Golden Triangle and Golden Crescent, moves from the northwestern and southwestern borders toward the coast [10], also adds pressure on HIV prevention. Finally, health care services in rural western China are insufficient - the quantity and quality of medical institutions are low, the service radius is large, the drug supply is scarce, and technical resources are limited [17].

**Table 1:** Construction of regional factors

Province	Category ^a^	Area (10,000 km^2^) ^e^	Population (million) ^f^	Proportion of rural population (%)^f^	Per capital annual net income of rural households (RMB) ^g, b^	Proportion of minority population (%) ^f, c^	Cumulative cases of HIV/AIDS (in thousands of cases) ^h, d^
Gansu	Province	42.58	25.99	64.1	3909.37(**B**)	9.43%(**c**)	1–5(**1**)
Guangxi	A.R.	23.76	47.96	60.0	5232.33(**B**)	37.18%(**b**)	50–100(**3**)
Guizhou	Province	17.61	35.30	66.2	4145.35(**A**)	36.11%(**b**)	10–50(**2**)
Inner Mongolia	A.R.	118.3	25.11	44.5	6641.56(**C**)	20.46%(**b**)	1–5(**1**)
Ningxia	A.R.	6.64	6.68	52.0	5409.95(**B**)	35.42%(**b**)	less than 1(**1**)
Qinghai	Province	72.23	5.88	55.3	4608.46(**A**)	46.98%(**b**)	1–5(**1**)
Shaanxi	Province	20.58	37.93	54.3	5027.87(**B**)	0.51%(**c**)	5–10(**2**)
Sichuan	Province	48.6	82.04	59.8	6128.55(**C**)	6.1%(**c**)	50–100(**3**)
Tibet	A.R.	120.22	3.24	77.3	4904.28(**A**)	91.83%(**a**)	less than 1(**1**)
Xinjiang	A.R.	166	23.60	57.2	5442.15(**B**)	59.9%(**b**)	10–50(**2**)
Yunnan	Province	39.4	47.42	65.3	4721.99(**A**)	33.37%(**b**)	100 and above (**3**)

^a^ A.R.: Autonomous Region

^b^ Regional rural residents’ economic status (RMB): **A.** Low-income area (less than 5000); **B.** Middle-income area (5000-6000); **C.** High-income area (6000 and above)

^c^ Regional ethnic composition: **a.** Han-dominated area (less than 10%); **b.** Mixed area (30–60%); **c.** Minority-dominated area (90% and above)

^d^ Regional HIV prevalence (in thousands of cases): **1.** Low-prevalence area (less than 5); **2.** Middle-prevalence area (5–50); **3.** High-prevalence area (50 and above)

^e^ The State Council of the People’s Republic of China. http://english.gov.cn/archive/

^f^ The Sixth National Population Census, 2010

^g^ China National Bureau of Statistics. China Statistical Yearbook 2012[M]. Beijing: China Statistic Press; 2012

^h^ Long Yuqin. Picture: Distribution map of provincial cumulative HIV/AIDS (number of deaths included) until Oct 2014. China HIV map: homosexual transmission accounts for more than 80%. http://news.ifeng.com/a/20141202/42619936_0.shtml

### Questionnaire design

For this study, we used a questionnaire written in Chinese, containing three sections. The first section was on sociodemographic information, including gender, age, education, occupation, and personal monthly income. Participants were classified into age groups of 16–40 years, 40–60 years, and above 60 years. Education was categorized into four levels – illiterate, primary, secondary, and tertiary, with reference to the revised 2011 version of the International Standard Classification of Education [25]. Occupation was classified according to the source of income, and personal monthly income classification was based on communication with local officials. The second section included 2 questions on HIV/AIDS: (1) Have you ever heard about HIV/AIDS (or do you know of the existence of HIV/AIDS)? (2) Please select all the transmission routes of HIV among blood, sex, mother-to-child, shaking hands, and droplets (multi-choice). Because implementation of the HIV health education relied on the general health education system, the third section of the questionnaire included 4 questions on access to health knowledge. Two questions were about direct access: (3) Please select the most frequently used channels to health knowledge based on your own situation among medical staff, mass media (broadcast, TV, newspapers, magazines, etc.), family members, and socializing (friends, colleagues, classmates, etc.) (multi-choice). (4) Did you participate in any health education activities in the last 6 months? Another two questions were on indirect access: (5) Have you had any physical examinations during the last year? (6) Have you had any non-communicable disease (NCD) during the last 6 months and, if so, did you receive any professional medical instructions? The 4 questions above were generated from previous studies [26–28] and adapted to our study setting. A pilot study was conducted with a small sample of the population before the main study was conducted.

### Sampling strategy

In every province, according to the gross regional product (GRP) ranking, three counties representing high, middle, and low economic status were randomly selected (3 × 11 = 33 counties). County GRPs were obtained from the statistics bureau. In 2011, more than 95% of rural residents participated in the New Rural Cooperative Medical System (NRCMS). Sick individuals could be reimbursed for 60–80% of their medical costs if they visited local public medical institutions. In the current Chinese health system, county-level medical institutions are the primary comprehensive hospitals, and most patients treated there are local residents. Since household surveys and telephone surveys were not practical due to geographic and language barriers, we performed a hospital-based questionnaire study at public county-level medical institutions. Normally, each county has 3 types of county-level public medical institutions, so that 99 were included (3 × 3 × 11 = 99 county medical institutions). Questionnaires were randomly distributed to patients at the selected medical institutions through a systematic sampling process according to their order of registration. Sample size at specific medical institutions was calculated according to its annual patient volume.

### Quality control

Questionnaires were administered by trained students from local medical colleges. Surveyors followed standardized procedures, with strategies to overcome the barriers of dialect, culture, and religion. Ethical approval was obtained from the University of Macau. All participants were fully informed of the research, and written informed consent was obtained. Additionally, the investigator could help explain the questionnaire; in some cases, the patients’ companions or medical staff could help overcome the barriers of dialect or ethnical languages.

### Statistical analysis

Descriptive statistics were used to define demographic characteristics. Chi-square analysis was used to analyse rate of awareness of HIV/AIDS (hereinafter, “HIV awareness rate”) among different subgroups. Through chi-square analysis, factors that significantly affected awareness of HIV/AIDS were put into a binary logistic regression equation by the conditional forward method, therefore allowing us to analyse the influence of access to health knowledge on HIV awareness. Chi-square was used to analyse the influence of access to health knowledge on awareness of HIV transmission routes. SPSS (Statistical Package for Social Sciences, Chicago, IL, USA), version 19.0, was used for statistical analysis. All statistical tests were two-sided, with a significance level of 0.05.

## Results

### Sociodemographic features

A total of 10,394 questionnaires were returned, and 9274 (89.2%) were valid. Among the 9274 participants, the age distribution quartile was 28, 40, and 53 years (range, 16–100) and 42.8% were male. Other sociodemographic features are shown in Table 2.

**Table 2:** Demographic features and Chi-square test for HIV awareness among rural residents in western China (*N* = 9274)

Variables	*N*	Proportion of population (%)	Rate of awareness (%)	*χ*^*2*^	*p* value
Patient type					
Inpatient	4345	46.9	80.8	0.079	0.778
Outpatient	4929	53.1	81.0		
Social-demographic variables					
Gender					
Male	3965	42.8	80.4	1.137	0.286
Female	5309	57.2	81.3		
Age					
16–40	4962	53.5	88.7	482.118^***^	< 0.001
41–60	2925	31.5	75.1		
above 60	1387	15.0	65.1		
Education					
Illiteracy	1304	14.1	60.0	853.465^***^	< 0.001
Primary	2233	24.1	70.5		
Secondary	4523	48.8	88.0		
Tertiary	1214	13.1	96.1		
Occupation					
Farmer	4284	46.2	74.0	340.533^***^	< 0.001
Migrant worker	1379	14.9	87.9		
Salary employee	1121	12.1	91.8		
Student	324	3.5	94.8		
Others	1338	14.4	86.3		
Unemployed individuals	828	8.9	75.6		
Personal monthly income					
No income	2621	28.3	74.1	186.448^***^	< 0.001
Less than 2000 yuan	4127	44.5	80.3		
2000–4000 yuan	1981	21.4	88.9		
4000 yuan and above	545	5.9	89.2		
Region					
Cumulative cases of HIV/AIDS					
Less than 5000	3458	37.3	77.1	59.513^***^	< 0.001
5000–50,000	2064	22.3	81.0		
50,000 and above	3752	40.5	84.3		
Per capita annual net income of rural households					
Less than 5000 *yuan*	3654	39.4	80.1	64.676^***^	< 0.001
5000–6000 *yuan*	3619	39.0	78.3		
6000 *yuan* and above	2001	21.6	87.0		
Proportion of minority population					
Less than 10%	3191	34.4	86.2	94.898^***^	< 0.001
30–80%	5512	59.4	78.5		
90% and above	571	6.2	74.1		
Access to HIV knowledge					
NCD (in the last 6 months) and medical instructions					
Do not have an NCD	6889	74.3	81.1	78.709^***^	< 0.001
Living with an NCD with medical instructions	1930	20.8	83.8		
Living with an NCD without medical instructions	455	4.9	65.7		
Had a physical examination (in the last year)					
Yes	3515	37.9	87.0	135.679^***^	< 0.001
No	5759	62.1	77.2		
Participated in health education activities (in last 6 months)					
Yes	2951	31.8	88.3	154.400^***^	< 0.001
No	6323	68.2	77.4		
Received health knowledge from medical staff					
Yes	4872	52.5	88.8	419.755^***^	< 0.001
No	4402	47.5	72.1		
Received health knowledge from the mass media					
Yes	6653	71.7	87.9	740.238^***^	< 0.001
No	2621	28.3	63.2		
Received health knowledge from family members					
Yes	795	8.6	76.2	12.189^***^	< 0.001
No	8479	91.4	81.3		
Received health knowledge by socializing					
Yes	1696	18.3	86.9	48.780^***^	< 0.001
No	7578	81.7	79.5		
Overall	9274	100.0	80.9	–	–

^***^P < 0.001

### HIV awareness

For the 9274 participants, HIV awareness rate (the rate of knowing of the existence of HIV) was 80.9% [95% CI: 80.1–81.7%]. Subgroup analysis was applied to regional factors, sociodemographic factors, and access to health knowledge. Three regional factors, regional rural residents’ economic status, regional ethnic composition, and regional HIV prevalence were constructed, respectively, by province-level of per capita annual net income of rural households, proportion of minority population, and cumulative cases of HIV/AIDS. Details are shown in Table 1. In the analysis of regional factors, participants’ HIV awareness rate increased with increase in cumulative cases of HIV/AIDS, from 77.1 to 84.3% (*χ*^2^ = 59.513, *p*<0.001). Per capita annual net income of rural households also had a positive influence on HIV awareness, with a cut-off point at 6000RMB (*χ*^2^ = 64.676, *p*<0.001). Simultaneously, awareness rate was lower in regions with more ethnic minorities, ranging from 86.2% in a Han-dominated area to 74.1% in Tibet (*χ*^2^ = 94.898, *p*<0.001), where more than 90% residents are minorities. Inpatient and outpatient status had no statistically significant effect on HIV awareness rates, and no difference was seen between the two genders. However, HIV awareness rate declined with increasing age, from 88.7% in young people (16–40 years) to 75.1% in middle-aged individuals (40–60 years), and 65.1% in the elderly (above 60 years) (*χ*^2^ = 482.118, *p*<0.001). In contrast, we noted an upward trend from 60.0 to 96.1% with participants’ educational levels increasing from less than primary education (the illiterate) to tertiary education (*χ*^2^ = 853.465, *p*<0.001), as well as from 74.1 to 89.2% with participants’ personal monthly income increasing from no income to RMB4000 and above (*χ*^2^ = 186.448, *p*<0.001). Three levels of HIV awareness rate were noted among different occupations (*χ*^2^ = 340.533, *p*<0.001): farmers and unemployed individuals (approximately 75%), migrant workers and others (approximately 87%), and students and salary employees (greater than 90%). Individually, the HIV awareness rates were 88.8, 87.9, and 86.9% of participants who received health knowledge from medical staff, mass media, or socializing, respectively, which were all greater than for those who did not. Participants who took part in health education activities and physical examinations both had a 10% higher awareness rate than those who did not. Conversely, those who received health knowledge from family members had a 5% lower HIV awareness rate than those who did not. We noted that only 795 participants belonged to this subgroup, accounting for 8.6% of the sample population. Participants who had an NCD in the previous 6 months and received medical instructions had the similar level of awareness (83.8%) as those who did not have an NCD (81.1%). In contrast, only 65.7% of participants who had an NCD but did not receive medical instructions knew of the existence of HIV. These results are shown in Table 2.

### Factors associated with HIV awareness

Except for patient type and gender, all demographic factors, regional factors, and methods of access to health knowledge were entered into a binary logistic regression model as dependent variables. HIV awareness rate was adopted as the independent variable. For demographic variables, participants who were 16 to 40 years old, illiterate, farmers, and had no income were the reference groups, respectively, for the categories of age, education, occupation, and personal monthly income. For regional variables, one variable - regional rural residents’ economic status - was excluded by forward conditional analysis. Low-prevalence area and low proportion of minority population were the reference groups, respectively, for regional HIV prevalence and regional ethnic composition. For access to health knowledge, the reference groups were those with individuals who did not receive health knowledge from medical staff, mass media, family members, and socializing; those who did not participate in health education activities; those who did not have a physical examination; and those who did not have an NCD. As shown in Table 3, compared with young people (16–40 years), middle-aged individuals (41–60 years) were less likely to know about HIV (OR = 0.533, 95% CI: 0.462–0.615, *p*<0.001) and older people (60 years and above) were even less likely (OR = 0.395, 95% CI: 0.329–0.474, *p*<0.001). Compared with farmers, students were more likely to know about HIV (OR = 2.469, 95% CI: 1.455–4.188, *p* < 0.01). Compared with the illiterate, those who had finished secondary education were more likely to know about HIV (OR = 2.275, 95% CI: 1.903–2.720, *p*<0.001), and having finished tertiary education increased this likelihood (OR = 4.519, 95% CI: 3.130–6.525, *p*<0.001). People who received health knowledge from medical staff (OR = 2.557, 95% CI: 2.244–2.914, *p*<0.001) and the mass media (OR = 3.812, 95% CI: 2.800–3.618, *p*<0.001) were more likely to know about HIV. Interestingly, people in high-prevalence areas and low-prevalence areas had the same likelihood of being aware about HIV (OR = 1.024, 95% CI: 0.882–1.188, *p* = 0.760), whereas those in middle-prevalence areas were less likely to know about HIV (OR = 0.722, 95% CI: 0.608–0.856, *p*<0.001). Finally, the likelihood of knowing of HIV decreased with an increased proportion of minorities (minority proportion according to province 30–80%: OR = 0.864; minority proportion according to province > 90%: OR = 0.453).

**Table 3:** Logistic regression results of HIV awareness among rural residents in western China (*N* = 9274)

Variables	OR	95% CI		*p* value
		Lower	Upper	
Social-demographic variables				
Age				
16–40	1			
41–60	0.533	0.462	0.615	< 0.001
Above 60	0.395	0.329	0.474	< 0.001
Education				
Illiterate	1			
Primary	1.340	1.137	1.579	< 0.001
Secondary	2.275	1.903	2.720	< 0.001
Tertiary	4.519	3.130	6.525	< 0.001
Occupation				
Farmer	1			
Migrant worker	1.137	0.928	1.394	0.215
Salary employee	1.346	1.017	1.783	0.038
Student	2.469	1.455	4.188	0.001
Others	1.349	1.093	1.666	0.005
Unemployed individuals	1.315	1.072	1.614	0.009
Personal monthly income				
No income	1			
Less than 2000 *yuan*	1.449	1.254	1.764	< 0.001
2000–4000 *yuan*	1.636	1.323	2.021	< 0.001
4000 *yuan* and above	1.412	1.011	1.971	0.043
Region				
Cumulative cases of HIV/AIDS				
Less than 5000	1			
5000–50,000	0.722	0.608	0.856	< 0.001
50,000 and above	1.024	0.882	1.188	0.760
Proportion of minority population				
Less than 10%	1			
30–80%	0.864	0.751	0.995	0.042
90% and above	0.453	0.347	0.591	< 0.001
Access to health knowledge				
NCD (in the last 6 months) and medical instructions				
Do not have an NCD	1			
Living with an NCD with medical instructions	1.218	1.023	1.451	0.027
Living with an NCD without medical instructions	0.689	0.542	0.875	0.002
Had a physical examination (in the last year)				
Yes	1.310	1.125	1.525	< 0.001
No	1			
Participated in health education activities (in last 6 months)				
Yes	1.413	1.204	1.658	< 0.001
No	1			
Received HIV knowledge from medical staff				
Yes	2.557	2.244	2.914	< 0.001
No	1			
Received HIV knowledge from the mass media				
Yes	3.812	2.800	3.618	< 0.001
No	1			
Received HIV knowledge from family members				
Yes	0.816	0.669	0.996	0.046
No	1			
Received HIV knowledge by socializing				
Yes	1.524	1.282	1.812	< 0.001
No	1			

### Relationship between access to health knowledge and awareness of HIV transmission routes

Table 4 shows the awareness rates of HIV transmission routes among different subgroups. When comparing the awareness rates of the three transmission routes among subgroups in each category of access to health knowledge, the mother-to-child route had the lowest level. Among all subgroups, the awareness rates of mother-to-child route were more than 10% smaller than the other two routes. In the aspect of subgroup analysis, low levels of awareness were showed of all three transmission routes among those who did not receive health knowledge from medical staff (blood, mother-to-child, sex: 56.5, 45.5, 56.3%, respectively) and mass media (blood, mother-to-child, sex: 48.5, 32.5, 46.1%, respectively). However, the awareness level of all three transmission routes was lower among those who received health information from family members (blood, mother-to-child, sex: 64.5, 53.5, 63.6%, respectively). Moreover, those who had an NCD without medical instructions also had low awareness levels; specifically, 51.0, 39.3, and 47.4% for the blood route, mother-to-child route, and sex route, respectively.

**Table 4:** Relationship between access to health knowledge and awareness of HIV transmission routes (*N* = 9274)

Access to health knowledge	N	Blood (%)	*χ*^*2*^	*p* value	Mother-to-Child (%)	*χ*^*2*^	*p* value	Sex (%)	*χ*^*2*^	*p* value
NCD (in the last 6 months) and medical instructions										
Do not have an NCD	6889	69.0	65.931^***^	< 0.001	57.2	57.474^***^	< 0.001	69.3	96.467^***^	< 0.001
Living with an NCD with medical instructions	1930	69.7			54.3			70.4		
Living with an NCD without medical instructions	455	51.0			39.3			47.7		
Had a physical examination (in the last year)										
Yes	3515	74.0	87.052^***^	< 0.001	58.9	23.497^***^	< 0.001	75.4	126.968^***^	< 0.001
No	5759	64.7			53.8			64.2		
Participated in health education activities (in last 6 months)										
Yes	2951	76.1	125.255^***^	< 0.001	61.4	56.120^***^	< 0.001	76.1	117.298^***^	< 0.001
No	6323	64.5			53.1			64.9		
Received health knowledge from medical staff										
Yes	4872	78.9	535.575^***^	< 0.001	65.0	356.523^***^	< 0.001	79.4	570.262^***^	< 0.001
No	4402	56.5			45.5			56.3		
Received health knowledge from mass media										
Yes	6653	76.0	656.038^***^	< 0.001	64.9	802.594^***^	< 0.001	77.3	846.813^***^	< 0.001
No	2621	48.5			32.5			46.1		
Received health knowledge from family members										
Yes	795	64.5	5.475^***^	0.019	53.5	1.845	0.174	63.6	9.326^**^	0.002
No	8479	68.6			56.0			68.9		
Received health knowledge by socializing										
Yes	1696	75.9	56.197^***^	< 0.001	64.0	57.762^***^	< 0.001	77.5	79.168^***^	< 0.001
No	7578	66.5			53.9			66.4		
Overall	9274	67.8	–	–	55.4	–	–	68.1	–	–

^**^P < 0.01, ^***^P < 0.001

## Discussion

The results revealed that the overall HIV awareness rate was 80.9% and we noted the following differences according to age group: HIV awareness rates were 88.7, 75.1, 65.1% among the young (16–40 years), middle-aged (41–60 years), and elderly (older than 60 years), respectively. Age was a suppressing factor for knowing of HIV/AIDS in a regression model. This result corresponded to a sharp increase in HIV infection among the Chinese elderly in recent years [12]. This phenomenon was partly attributed to older men’s engagement in commercial sex and older women’s participation in extramarital sex, with poor knowledge of HIV [29]. Moreover, HIV health education for the elderly was neglected in the early years [29–31]. Thus, there was a need to improve HIV health education for this age group. The HIV awareness rate for farmers or unemployed individuals was approximately 75%, which was about 10% less than for migrant workers and about 20% less than for students and salary employees. Personal monthly income had a positive effect on HIV awareness, but more than two thirds of participants had a personal monthly income of less than 2000RMB. When occupation and personal income were considered together, we found that those with the lowest income may need special attention. In the current economic structure of China, rural residents often leave home yearly for better wages as migrant workers. In comparison, those who remained unemployed or who stayed to work on farms fared worse in the labour market. Living in remote villages, they had insufficient health care resources and access to health information. Furthermore, considering the different categories, poor farmers and the unemployed, residents with no income, and those who had an NCD without professional medical instructions, had a great overlap, and might be a same group of people. This small population may be the most impoverished, and more aggressive strategies should be taken to increase HIV awareness in this group, such as in-person education.

As to awareness of the routes of HIV transmission, sexual transmission and blood transmission were at similar awareness levels in each subgroup, out of all the categories classified according to access to health knowledge. In contrast, awareness of mother-to-child transmission was more than 10% lower than the other two routes in each subgroup. This finding was similar to those of other studies in the same region [32]. Other studies showed that China’s program for preventing mother-to-child transmission (PMTCT) was effective on the basis of adequate publicity [1], and some cases of PMTCT failure were attributed to lack of knowledge of HIV and the PMTCT program [33, 34]. The mother-to-child transmission route and the PMTCT program deserve more publicity.

Our results were consistent with previous research which showed that residents mainly rely on traditional sources of health knowledge in rural western China, such as medical personnel, radio and TV, magazines, and leaflets [17]. In this study, residents who received health knowledge from medical staff had the best results on questions about HIV transmission, and this result revealed that efforts to implement HIV health education relied too heavily on medical staff. The second source of information was mass media, which was also the most efficient. This result was consistent with other studies [17, 26], and called for mass media to take more responsibility in HIV health education and prevention. The third source of information was socializing. This finding was consistent with another result of this research which indicated that better performance was seen among those who had engaged in health education activities. However, due to the remote setting, it is not an efficient strategy to encourage frequent socializing and participation in activities in this area. Finally, only 8% said that they gained health knowledge from family members. The HIV awareness rate and the awareness rates for all three transmission routes among this small subgroup were more than 10% lower than the rates for the majority, which exposed a low level of health literacy in this area at the family or household level.

As mentioned above, this research indicated that the elderly and the most impoverished populations needed special attention in an individual aspect. Geographically, this research showed that regional economic development had little impact since the variable of regional rural residents’ economic status was excluded from the regression model. Regions with complex ethnic compositions deserve more attention since the proportion of minority populations was a suppressing factor for HIV awareness. From a longitudinal viewpoint, it was still an issue of how to elevate the population’s literacy level by educating the minorities within the community [35]. The results of this investigation also showed that education was the strongest factor promoting residents in rural areas to know about HIV and, hence, education was a key determinant of HIV awareness in the context of rural west China. Both general education and HIV health education were important [36]. Improving the general education level could diminish HIV discrimination, as well as enhance the ability of people of low socio-economic status to understand health information [37, 38]. However, it was impossible to improve the level of general education at one strike in such a complicated area. Health education was more direct in cultivating behavioural changes concerning health and to improve health literacy. Chinese government regarded the systematic health education in schools as one of the key strategies to improve the health literacy of general population, anticipating the students to spread the right knowledge to their family members and communities [39]. On this basis, apart from multiple publicity access, the Chinese government strengthened HIV health education in schools [6]. Although few investigations focused on the effects of such a strategy on the family, it was rational to infer that educating the children would raise the awareness of parents through policy promotion and parent-child interaction. During the past several years, the Chinese government has mobilized university students to play an active role in HIV health education in a series of voluntary HIV prevention programmes [1]. Minority university students, especially those growing up in minority communities, had huge advantages in HIV education activities [40]. On this basis, the strategy of encouraging students to spread their knowledge of HIV at their homes or back in their hometowns should be strengthened.

### Limitations

There were three major limitations to this study. First, all participants were patients at local public hospitals, which could introduce a bias from being in contacting with medical institutions. Second, the main source of health knowledge was general, especially mass media. More specific access to the source of information, for example through journals, broadcasts, online resources, or mobile devices, was not analysed. Third, demographic factors, regional factors, and access to health information were analysed individually, whereas empirically they were not mutually exclusive and their collaborative effects should be considered [41].

## Conclusion

HIV health education covers 80% of the rural population in western China, and more active health education measures are needed for the elderly, the most impoverished population, and the minority community. Mass media should take greater responsibility in HIV health education for the general population. The mother-to-child transmission route called for special attention. In the long run, the strategy of delivering HIV knowledge through school students to their original communities and family members should be continuously encouraged. Strengthening education programs could have great importance in fostering HIV awareness.

## Data Availability

The datasets used and/or analyzed during the current study are available from the corresponding author on reasonable request.

## Abbreviations

AIDS: Acquired immune deficiency syndrome
CSW: Commercial sex worker
GRP: Gross regional product
HIV: Human immunodeficiency virus
IDU: Intravenous drug user
KAP: Knowledge-attitude-practice
NCD: Non-communicable disease
NRCMS: New rural cooperative medical system
PMTCT: Prevention of mother-to-child transmission
SPSS: Statistical package for social sciences
STD: Sexually transmitted disease
WDP: Western development program
